# Fighting falsified medicines with paperwork – a historic review of Danish legislation governing distribution of medicines

**DOI:** 10.1186/s40545-016-0078-2

**Published:** 2016-10-06

**Authors:** Rasmus Borup, Susanne Kaae, Timo Minssen, Janine Traulsen

**Affiliations:** 1 Department of Pharmacy, University of Copenhagen, Universitetsparken 2, 2100 Copenhagen, Denmark; 2 Centre for Information and Innovation Law, University of Copenhagen, Studiestræde, 1455 Copenhagen, Denmark

**Keywords:** Harmonisation, Legislation, European Union, Falsified Medicines Directive, Enforcement

## Abstract

**Background:**

Many areas of pharmaceutical legislation in the European Union (EU) are harmonised in order to promote the internal market and protect public health. Ideally, harmonisation leads to less fragmented regulation and cross-border complexities. This study, however, focuses on an increasingly harmonised legislative area that is subject to increases in requirements and complexities: the distribution of medicines. This study compared Danish legislation governing the distribution of medicines before and after Denmark joined the EU in order to assess the impact of EU harmonisation, as well as to evaluate whether the drastic increases in requirements mandated by the Falsified Medicines Directive of 2011 correspond to a new approach to governing the pharmaceutical supply chain.

**Methods:**

A review was conducted of 115 applicable Danish laws, executive orders and guidelines from 1913 to 2014. Legal requirements were organised according to the year they were published and the companies they affected. Greater changes in legislative requirements were developed through inductive content analysis.

**Results:**

Early legislation positioned pharmacies as gatekeepers, requiring them to identify and stop medicines of substandard quality. Legislation to regulate the supply chain was slow to materialise. After Denmark joined the EU, the scope of legislation widened to include all actors in the supply chain, and the quantity of legislation increased dramatically. Simultaneously, requirements became more specific, thereby promoting a formalistic interpretation and focusing the attention of companies and authorities on predefined areas with little room to implement innovative solutions. Over time, documentation became the focus of legislation, requiring companies to provide documentary evidence for their compliance with legislation. The Falsified Medicines Directive continues these trends by increasing requirements for documentation and promoting a formalistic interpretation.

**Conclusion:**

The legislative approach adopted since Denmark joined the EU gives companies and medicine inspectors little room to interpret legislation. The Falsified Medicines Directive does not depart from this approach. Legislation seems more focused on enforcing similar requirements than on benefiting public health. Legislation may benefit from allowing room for local interpretation of requirements.

**Electronic supplementary material:**

The online version of this article (doi:10.1186/s40545-016-0078-2) contains supplementary material, which is available to authorized users.

## Background

The European Union (EU) ensures that the quality, efficacy and safety of pharmaceuticals are regulated by similar legislation in all EU countries. Pharmaceutical legislation within the EU is harmonised to allow medicines to travel between EU countries with a minimum of barriers and to safeguard public health [1, 2]. As a result of harmonisation, EU member states have limited autonomy over the pharmaceutical legislation in their own country [3]. The EU claims that the harmonised rules contribute to a high level of safety for consumers [4]. However, others point out that breaking down trade barriers is in effect a deregulatory action likely to have negative effects on public health [5, 6].

Researchers who argue that harmonisation lowers requirements tend to focus on requirements relating to the development of medicines and to pay less attention to legislation governing manufacture and distribution [7–12]. Although research in the regulation of medicines’ development is important, there may be something to be learned from looking at other areas of legislation, such as The Falsified Medicines Directive (the directive). Published by the EU in 2011, the directive aims to protect the public from falsified medicines. The directive imposes strong controls on the supply chain in order to keep falsified medicines out of European pharmacies. These measures have significant ramifications for supply chain actors [13–15], and some stakeholders, including manufacturers and medicine authorities, have argued that the directive raises the bar too high [16, 17]. As such, the directive does not seem to mirror the trend that harmonisation leads to deregulation.

In an effort to study the effects of EU harmonisation on the distribution of medicines, an area often ignored by researchers, this study analysed pharmaceutical legislation in Denmark before and after the country joined the European Community in 1973. Denmark is a small EU country with traditionally high regulatory standards, strong enforcement, a low level of corruption and a long history of pharmaceutical production.

Based on an historical review of legislation, this paper identifies characteristics in the developments in Danish legislation on the distribution of medicines, paying particular attention to changes after the Danish enrolment in the EU. In this context, this study examines whether the measures adopted in the directive introduced a new approach to governing the pharmaceutical supply chain.

## Methods

This study used legislation governing the distribution of medicines as its empirical material. Although there are substantial and formal differences between laws, executive orders and guidelines, these differences will be ignored in this article. The most effective argument authorities have for enforcing rules in the pharmaceutical sector – regardless of whether these rules are written in a law, an executive order, or a guideline – is the threat of revoking a company’s license to operate. This threat is only used when a company is overall non-compliant with the rules. However, rules written in laws, executive orders and guidelines all matter in the assessment of the compliance of a company, and they will therefore be referred to collectively as legislation and treated equally in this article.

It was decided to analyse past Danish legislation in order to answer the research question. As the first Danish Pharmacy Act was published in 1913, and due to the Danish tradition of manufacturing medicines locally at pharmacies, it was expected that distribution of medicines would be nearly non-existent in the early 1900s.

A documentary search of legislation from 1913 to the summer of 2014 was conducted to identify legislation governing the protection of medicines during distribution. Four different types of government publications were manually searched for relevant legislation at the Faculty of Law Library, Copenhagen: Proceedings from the former and current Parliament (*Rigsdagstidende* and *Folketingstidende) as well as adopted laws* (*Lovtidende)* and executive orders (*Ministerialtidende)*. Legislation published after 1985 was available through the government website, www.retsinformation.dk. Danish legislation is indexed according to topic of legislation. The keywords used to identify legislation for this study were “pharmacy” (*apotek*), “medicines” (*medicin*), “pharmaceuticals” (*lægemidler*), and “health” (*sundhed*). Legislation was identified using the keywords and read by the first author before deciding whether to include it in the analysis. Documents were excluded if they did not relate to labelling, storage, distribution or manufacture of medicines. Documents were also excluded if they related only to specific types of medicines (narcotics, veterinary medicines, medical gasses), or, if reading determined that documents related only to manufacturing processes, pharmacy price setting or similar areas outside the scope of the study. When legislation referenced other documents (e.g. the pharmacopeia or EU guidelines), the referenced documents were located via the library or websites and included in the analysis. A total of 115 documents were identified as being relevant. Each document was then registered along with the year it was published (see Additional file 1).

### Analysis

The documents underwent a three-step content analysis [18]. First, categories relating to handling of medicines during distribution and storage were inductively developed through thorough reading of the documents. Categories were developed to filter out legislative requirements related to reimbursement, ownerships of pharmacies, and other areas irrelevant to this study. A total of six different categories were developed: pre-purchase screening, detection (including complaints handling), stop and recall (including traceability), quality maintenance, preventing unauthorised handling, and management. See Table 1 for an explanation of each category. The categories were developed by the first author, presented to two Danish experts in legislation on distribution of medicines, and adjusted according to their feedback.

**Table 1:** Categories of ‘Quality Requirements’

Category of 'Quality Requirements'	Purpose of 'Quality Requirement'
Pre-purchase screening	To pre-qualify suppliers; evaluation of potential suppliers.
Detection (including complaints handling)	To evaluate the quality of received goods.
Stop and recall (including traceability)	To ensure that distributed products thought to be substandard or falsified are stopped or effectively and swiftly recalled.
Quality maintenance	To maintain the quality of the product while in the company’s care.
Preventing unauthorized handling	To prevent products from moving into the illegal supply chain.
Management	To ensure that company activities are performed satisfactorily.

The first five categories of ‘Quality Requirements’ all relate to physical handling of medicines or preventing the distribution of substandard or falsified medicines. The category ‘Management’ encompasses requirements not related to the physical handling of medicines and not directly affecting the quality of the medicines, but rather the management of such activities. Although requirements in the category ‘Management’ do not directly affect the quality of medicines, this category was included in the analysis as it was clear even prior to the analysis that legislation often uses management tools to regulate the distribution of medicines.

Second, the documents were scrutinised by deductively identifying legislative requirements related to the categories and transferred to tables and organised according to the year published and the actors to which they applied. Third, tables were scrutinised by the first author and greater changes in requirements signalling a change in scope or adoption of new approaches were identified and validated via discussions with co-authors. Particular attention was given to the difference between the periods before and after Denmark joined the EU.

## Results

**Fig. 1:**
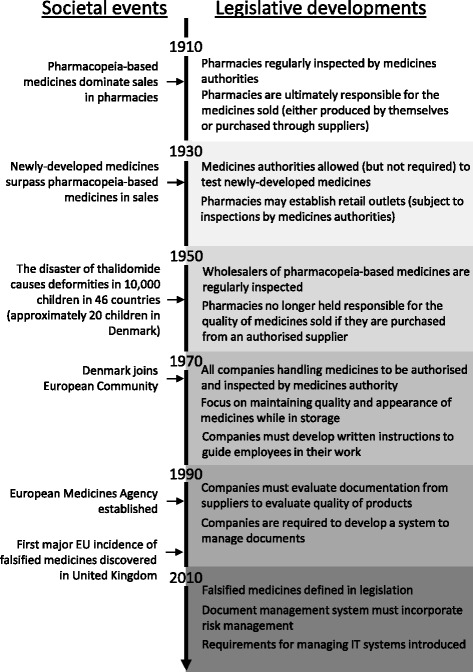
Timeline of the developments in Danish legislation governing distribution of medicines

The first Pharmacy Act was published in 1913, replacing the existing executive order from 1672. At this time, most medicines were manufactured locally at pharmacies from recipes published in the pharmacopeia. However, the industrial revolution was beginning to have an impact on the sale of medicines in Denmark. Pharmacies were able to buy both newly-developed (industrially-produced medicines) and traditional pharmacopeia-based medicines through foreign and domestic factories [19]. However, only pharmacopeia-based medicines were regulated in the beginning (see Fig. 1 for overview of developments).

Prior to the Danish enrolment in the European Community in 1973, three Pharmacy Acts were published approximately every 20 years. They focussed on pharmacy management and employee education as the most important tools to ensure high quality medicines, but provided very few details on how pharmacies should operate. Domestic factories and importers were also included in the scope of legislation, but they received similarly unspecific instructions. Common for all companies included in the legislation was the stipulation that they were subject to regular visits from health authority medicine inspectors. As pharmacies could only purchase pharmacopeia-based medicines from other pharmacies or domestic factories or importers, the supply chain was kept short and simple. Pharmacies were held responsible for the quality of any pharmacopeia-based medicine they sold, regardless of whether they manufactured or purchased the medicines [19–21].

Although the sales of newly-developed medicines had surpassed pharmacopeia-based medicines during the 1940s [22], legislation to regulate newly-developed medicines was slow to materialise. The measures taken to protect the quality of pharmacopeia-based medicines were not automatically applied to newly-developed medicines. For instance, companies manufacturing or distributing newly-developed medicines were not usually subject to legislation [21].

Denmark joined the EU (known as the European Community at the time) in 1973. The process of harmonising legislation with other member states fostered a new all-encompassing approach to regulating the manufacture and distribution of medicines. With the Medicines Act of 1975, legislation centred on the medicines instead of the pharmacy. The traditional distinction between pharmacopeia-based and newly-developed medicines ended. All companies physically handling medicines, whether through manufacture, storage, distribution or sale, were to be authorised and monitored through regular visits from medicine inspectors, resulting in an increase in the number of companies subject to legislation (see Table 2) [23].

**Table 2:** The expansion of actors in the supply chain subject to legislation before Danish enrolment in the EU (left) and after enrolment (right)

Companies subject to Legislation in 1972 (prior to EU accession)	Companies subject to legislation in 2014 (after EU accession)
Pharmacies Manufacturers Retail outlets Wholesalers (for pharmacopeia-based medicines)	Pharmacies Manufacturers Retail outlets Wholesalers (for all medicines) Parallel importers Retail shops Internet shops Brokers Distributors of active pharmaceutical ingredients

**Fig. 2:**
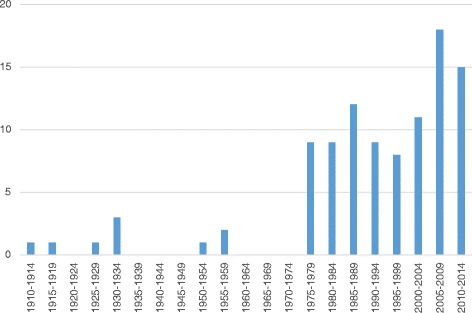
The number of published pieces of legislation from 1910 to 2014 related to the distribution of medicines

**Fig. 3:**
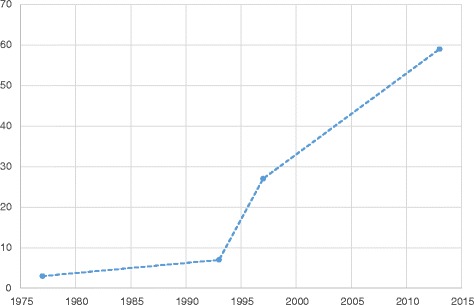
The number of required documents for wholesalers

The quantity of legislation rose remarkably after Denmark joined the EU (see Fig. 2). The rise in quantity correlated with increasing specificity of legislative requirements, initially in 1977 by establishing requirements for the layout and cleaning of storage areas in order to maintain the quality of medicines. The same year, legislation began to require companies to produce written instructions and to record certain activities related to the handling of medicines. The focus on documentation and specificity of legislation continued, in 1997 resulting in the requirement to establish a document management system as well as written procedures describing document handling activities. The requirement for producing documentation today encompasses risk assessments of delivery routes, validation reports, auditing reports, training reports, qualification reports, corrective and preventive action reports, temperature evaluation reports, etc. As shown in Fig. 3, the number of different documents (procedures, records, descriptions, evaluation, etc.) required by legislation has risen dramatically since 1977.

### The falsified medicines directive

The directive was partly implemented in Danish legislation in 2013, most notably introducing requirements for a new type of company, brokers, which have no physical contact with medicine, and expanding the regulated supply chain to include distributors of active pharmaceutical ingredients. The main component of the directive has yet to be implemented: by 2019, all pharmacies will be required to verify the authenticity of medicines before dispensing them by scanning a unique barcode printed on each package of medicine. The scanner will verify that the unique barcode is genuine by checking an EU-wide database accessible only to pharmaceutical manufacturers, wholesalers and pharmacies [24]. Data will be stored for later review by medicine authorities.

Although the specific focus on falsified medicines is recent, the requirement that pharmacies should only dispense medicines of good quality is by no means new. The directive may appear drastic to some stakeholders, but it continues the trends observed in Danish legislation since Denmark joined the EU, in particular by focusing on documentation and adding further specificity to requirements.

## Discussion

Danish legislation was harmonised with EU member states in response to the Danish enrolment into the EU in 1973. Legislation has since continued to develop according to three main principles identified in this study: 1) Legislation has expanded to cover more types of actors, 2) legislative requirements have become increasingly specific, 3) documentation has gained increasing importance. The consequences of these developments are discussed below.

### More supply chain actors

The inclusion of different types of supply chain actors presents challenges, as requirements are rarely the same for all. Our data show that separate pieces of legislation are published to cover specific types of actors. Therefore, no one piece of legislation or one set of requirements applies to all actors in the supply chain. However, actors in the supply chain interact with each other, and legislation needs to regulate their interactions in a way that ensures the safety of medicines. For example, it is important to have a clear agreement on who is responsible for the quality of medicines during transport, the supplier or the purchaser. Similarly, it is important to make sure that a customer complaint made at a pharmacy is forwarded to the correct pharmaceutical company for further investigation. This study found that the legislation regulating the supply chain as a whole has become highly complex. Complexity inevitably makes compliance more demanding and is already forcing companies to hire experts in regulatory requirements [25]. As the Organisation for Economic Co-operation and Development suggested, resources might be put to better use by eliminating some of the legislative complexity [26].

### Specific requirements

The increased specificity of legislation inevitably leads to a more formalistic interpretation of the rules, which can have both positive and negative consequences. On the one hand, specific requirements provide a clear checklist for authorities when assessing the compliance of companies during inspections, allowing them to focus on areas predefined as being the most important [27]. This also translates positively to companies, as they are more likely to know whether they are in compliance with requirements prior to inspections from authorities [28]. Specific requirements may also make compliance easier for some companies, as the important areas requiring attention and the level of attention required have already been identified and described in legislation [29].

On the other hand, some companies may want to focus on other areas or use strategies other than those prescribed by legislation. Companies may even want to change the way they handle medicines entirely. Such preferences are difficult to accommodate when legislation sets specific requirements [30]. Specific legislation therefore carries the risk that innovative and essentially better or less costly ways of performing tasks will not be implemented, an argument supported by previous studies on regulation of pharmaceuticals [31, 32].

### Documenting instead of handling medicines

As shown in the analysis, recent requirements focus attention on documentation. By requiring companies to produce documentation of their compliance, medicine inspectors are able to discover events of non-compliance that happened in the past [33]. But as Power [34] describes, this type of compliance monitoring has a tendency to focus attention on the system of control rather than on the company’s first order of business.

Companies are required to produce documents that describe most activities and to provide records to document the execution of these activities. Companies are required to establish elaborate management systems to manage the documents, as well as allocating employees to assess and maintain the systems. The resources that companies expend on complying with the requirements for document creation and maintenance are most likely considerable and could potentially be put to more productive use.

### Harmonisation with enforcement in mind

This study found no signs of the deregulation typically reported when describing the effects of harmonising pharmaceutical legislation. On the contrary, requirements have increased following the Danish enrolment into the EU.

The more recent developments identified in this study promote a uniform enforcement of requirements: specific requirements promote the enforcement of similar standards in all member states, and the focus on documenting compliance deters non-compliance. Prior to joining the EU, Danish legislation was not focused on enforcement. Medicine inspectors would be unlikely to discover non-compliance, as requirements were broadly formulated and companies were not required to keep records of their activities. Enforcement therefore seems to have gained increasing importance in Denmark since joining the EU.

Although enforcement is obviously important, enforcement should not be the primary goal of legislation. Compliance monitoring should be performed using a minimum of resources, thereby allowing companies to focus on supplying medicines cheaply and timely while maintaining their quality. Further, legislation should allow room for companies and medicine inspectors to adopt the specific measures most suitable for promoting public health. Enforcing specific requirements with little view to the overall goal of legislation, protecting public health, may provide little value to patients.

This is exemplified in the directive that requires only prescription medicines to carry unique barcodes, thereby exempting non-prescription medicines. Although logically, voluntary use of unique barcodes on non-prescription medicines would only enhance the protection of public health, the European Commission, responsible for the legislation, has insisted on a formalistic interpretation of the directive and has refused to allow unique barcodes to be added to non-prescription medicines, even on a voluntary basis [35].

Hindering use of unique barcodes on medicines might ultimately lead to the consumption of falsified medicines by consumers. Such formalistic interpretation allows companies and EU member states little room to interpret legislation to fit local settings, even if such interpretation benefits public health [36].

The focus on enforcing similar requirements may be necessary for the EU to ensure the well-functioning of the internal market. However, it may be beneficial to adjust legislation and allow companies and medicine inspectors more discretion in implementing legislation. Recent pharmaceutical legislation may be too focused on enforcing harmonised requirements, rather than on making sure that requirements benefit public health. A similar view has been presented by Permanand [37].

The developments observed corresponds with Wards’ description of New Public Management, where trust is “replaced by assessment at a distance” [38]. As Abraham has previously suggested that New Public Management has shaped pharmaceutical legislation [39], the developments observed in this study may benefit from being analysed in such a context.

## Conclusions

This study did not find that Danish harmonisation with the EU caused deregulation. It did find, however, that the focus of harmonised legislation to enforce similar requirements for all might have unintended side effects. Rather than allowing companies and medicine inspectors to focus on protecting public health, harmonised legislation tends to focus attention on compliance with requirements that do not always fit the situation. This is exemplified by the Falsified Medicines Directive. There seems to be a risk that the overall goal of legislation, to protect public health, could become secondary to the efforts to ensure equal compliance among companies.

Keeping in mind that the protection of public health is an important goal of member states, we propose that legislation allows companies and medicine inspectors the possibility to interpret legislative requirements in order to make decisions that benefit public health.

## Additional file

Additional file 1: List of legislation. (XLSX 18 kb)

## Abbreviations

EU: European Union
